# Supplementary material for: Patterns of Intron Gain and Loss in Fungi
Source: PLoS Biol. 2004 Nov 30;2(12):e422. doi: 10.1371/journal.pbio.0020422 (PMC532390; doi:10.1371/journal.pbio.0020422)
Supplement: Table S1 — Also available at http://genes.mit.edu/NielsenEtAl/. (4.3 MB ZIP). [file pbio.0020422.st001.zip › NielsenEtAl/html/323.html]

AN2450.1.NCU03526.1.MG06733.1.FG06030.1


```
 CLUSTAL W (1.82) Multiple Sequence Alignments - Introns Inserted


Sequence 1: AN2450.1	610 aa
Sequence 2: FG06030.1	679 aa
Sequence 3: NCU03526.1	641 aa
Sequence 4: MG06733.1	728 aa
Alignment Length: 750 aa
Number Identitical Residues: 271 aa
Alignment Score (without introns) 13034


MG06733.1 	MATNPESPLPIHNPPPIANAAPQPAPPAQPMQPAQHVTQR------QVVQPTPRTRDTFN
NCU03526.1	-------------------------------------------------MTSQLTAEAVL
FG06030.1 	MDTTSADQAPAQTQLATASSAAAPVPAQQPQTQQQAQKQTVSSENAPQRRPPTITRDRHN
AN2450.1  	-----------------------------------------------------MTRDTYL
          	                                                      * :   

MG06733.1 	GQALGRE---TNARVKQ0ARVLMVGAGGIGCELLKNLALAGFGEIHAVDLDTIDLSNLNR
NCU03526.1	ALAPAETPETLLTTTSQ~ARVLMVGAGGIGCELLKNLVLTGFGEVHVVDLDTIDLSNLNR
FG06030.1 	QQSLGAS---LNTSIKQ0ARVLMVGAGGIGCELLKNLVLTGYGEIHIVDLDTIDLSNLNR
AN2450.1  	KRSLGTQ---ANR-IRE0SRVLLVGAGGIGCELLKNLLLTGFGEIHVIDLDTIDLSNLNR
          	  : .           : :***:************** *:*:**:* :************

MG06733.1 	QFLFRHEHIKKSKAK~VAKEAAQKFNPNVKIEAHEANIKSPQFNVEWFRSFAVVFNALDN
NCU03526.1	QFLFRHEHIKKSKAL~VAKEAAQKFNPAVKIVAHHANIKDAQFNIEWFSSFRIVFNALDN
FG06030.1 	QFLFRHEHIKKSKAL0VAKEAAQRFNPNVKIVAHHANIKDDQFTVAWFQQFRIAFNALDN
AN2450.1  	QFLFRHEHIKKPKAI0VAKEVAQKFQPSARIEAYHANIKDSKFDVDWFATFNVVFNALDN
          	***********.**  ****.**:*:* .:* *:.****. :* : **  * :.******

MG06733.1 	LDARRHVNRMCLAADVPLIDSGTTGFNGQVQVTKK~GVTACYDCEPKDPPKSFPVCTIRS
NCU03526.1	LEARRHVNKMCLAADVPLIESGTTGFNGQVQVIKK~GVTACYDCAPKETPKSFPVCTIRS
FG06030.1 	LEARRHVNKMCLASDVPLIESGTTGFNGQVQVIKK~GVTACYDCTPKEAPKSFPVCTIRS
AN2450.1  	LDARRHVNMMCLAADVPLIESGTTGFNGQVQVIKK0NVTECYDCNSKEVPKSFPVCTIRS
          	*:****** ****:*****:************ ** .** **** .*: ***********

MG06733.1 	TPSQPIHCIVWGKSYLLN~EIFGTSE-DESAFDHSADQNNA~EEVAELKREALALRAIRD
NCU03526.1	TPSQPIHCIVWGKSYLLN~EIFGASE-DESAFDHTVDGDNA~QEIEELKRESAALRKIRN
FG06030.1 	TPSQPIHCIVWGKSYLLN2EIFGTSE-DQAAFDHSTDADNA1KEIEELKKESAALKQIRD
AN2450.1  	TPSQPIHCIVWAKSYLLP2ELFGTSETDTEEFDYSADADNV1EEIENLQREARALKEIRQ
          	***********.*****  *:**:**:*   **::.* :*. :*: :*::*: **: **:

MG06733.1 	SIGTDKFPQMLFDKVFKADVERLRSMTDMWKDRKPPTPLDFETLKTKSAEDLNRTDATLK
NCU03526.1	SVGTEEFAQMLFEKVFKTDIERLRSMEDMWKTRKPPEPLNYKELLDKAKSLDK--DKVLK
FG06030.1 	ATGTSEFQQMLFDKVFNADIERLRSVEDMWTSRRAPEPLKYETVLAQASDAMANKKMLLE
AN2450.1  	SMGSAEFAQKVFDKVFKEDINRLRGMEDMWTSRKAPEPLDFKELEGTLSTVEP--EVSLK
          	: *: :* * :*:***: *::***.: ***. *:.* **.:: :           .  *:

MG06733.1 	DSQRLWSLEENFSVFIDS~LSRLSKRILELRKSKTPESPEPIIEFDKDDQDTLDFVASSA
NCU03526.1	DAQKVWSLEENLVVFNDS~LERLSKRVLENKS--AG--EESIITFDKDDEDTLDFVAASA
FG06030.1 	DDQRVWSLEESLVVFNDS~LDRLSKKILELKKNKASEDSEPTLSFDKDDIDTLDFVTASA
AN2450.1  	D-QRVWTVSENLAVFKDS2LDRLSKRLKTLQSEESG--SPAVLVFDKDDVDTLDFVTASA
          	* *::*::.*.: ** ** *.****::   :...:     . : ***** ******::**

MG06733.1 	NIRSHIFGIEGKSLFDIKQ~MAGNIIPAIATTNAIVAGLCVLEAFKVLKGQYEAAKE0AF
NCU03526.1	NIRSAVFGIDRKSKFDIKQ~MAGNIIPAIATTNAIVAGLCVLEAFKVLKGHYEQAKE~VF
FG06030.1 	NIRSHIFCIDKKSRFDTKQ~MAGNIIPAIATTNAIVAGLCVLQSYKVLKGEYAQSKE~VF
AN2450.1  	NLRATIFGIEPKSKFDTKQ1MAGNIIPAIATTNAMTAGLCVLQALKVLKGDYDHAKM0VF
          	*:*: :* *: ** ** ** **************:.******:: *****.*  :*  .*

MG06733.1 	LTPFRETGRIGIDRPRKPNPDCPVCSVFQISVVADLSRATLDNLVE-FVKHGVGFGEKDF
NCU03526.1	LTPFANARMLASDKSREPNPDCPVCGVYQTRAYVDLEKATLNDLVEHLIKTNLGYGEKDF
FG06030.1 	LTPFAAARLLAPDRSREPNPDCPVCSVYFTSVVTDLSRATLKDFVDEIVKSKLGYEGKEF
AN2450.1  	LE-RSGARAINSESLNPPNPHCPVCSVAHARIEIDLTRATLNDLVENILRTQLKY-GQEF
          	*     :  :  :  . ***.****.*       ** :***.::*: :::  : :  ::*

MG06733.1 	SVHVQDGKDLKMLYD---VEETENLPKLLTELG1VSPDSFVTIMDDDDDGLVNVVILIEA
NCU03526.1	AISNEVG----ILYD---PDETDNLEKKLSELG1IKSDSFLTITDEDDEEPFVNVVVAIQ
FG06030.1 	VVNNDVG----TLFECFEDGDDENLPKKLSDLG1IKKDSFLTIIDQDDEDTFVNVVINIQ
AN2450.1  	SVNTEQG----TIYD---PDLEDNLPKKLSDLG~ITTSAFLTVIDEDEQPRVNLQLIVVA
          	 :  : *     :::       :** * *::** :. .:*:*: *:*::  .   ::   

MG06733.1 	P2K~TADDNASAIQALHPDKKLDIPKKPAKKPPQPPTSN------GAVVSNGTSLTLE--
NCU03526.1	E~A2KEPLGDKPVKGILDPEDVKIPLKPKKQSQPEPVATPTAATNGASISNGQNGGVINL
FG06030.1 	E~G2TLDGDKKPVEAKFS----DKPEIPRRPKKPQPAE---------SNSNGETNGEA-V
AN2450.1  	P~G~SPPSEEQPIVLNR------IPEIPRKPQAPAPYD---------AEANGTSN----L
          	    .     ..:           *  * :     *             :** .      

MG06733.1 	GDNEKTQKNPKRPREGGEADEAANGREPKKAKIRGGDDDVVVVTDEGGAL0LSTRTSGWM
NCU03526.1	DGDEPMTTPAKSLKRGHPEDAEGPSVKKIKANDKAADDDIVFIEDSAGAI~VIDDD----
FG06030.1 	DAEHKGVKRLHTEDGGQPLKKVKITPPGAEIVDVDEDQNEGHANG--GAI~VIDD-----
AN2450.1  	GKRKRDAN--ETELNGDPPTKRVANVSISDGADKAHPIDLSEAEG--GAI~LIDDD----
          	.  .   .  .    *             .        :     .  **: :        

MG06733.1 	HISNRAQSSIEIENAAMCIRVGGQKESGIGYLLGRAHPAEGY
NCU03526.1	------------------------------------------
FG06030.1 	------------------------------------------
AN2450.1  	------------------------------------------
          	
```
